# Long noncoding RNA expression profiles in gut tissues constitute molecular signatures that reflect the types of microbes

**DOI:** 10.1038/srep11763

**Published:** 2015-06-30

**Authors:** Lunxi Liang, Luoyan Ai, Jin Qian, Jing-Yuan Fang, Jie Xu

**Affiliations:** 1 State Key Laboratory for Oncogenes and Related Genes; Key Laboratory of Gastroenterology & Hepatology, Ministry of Health; Division of Gastroenterology and Hepatology, Renji Hospital, School of Medicine, Shanghai Jiao Tong University, Shanghai 200001, China; 2 Shanghai Cancer Institute, Shanghai Institute of Digestive Disease, 145 Middle Shandong Rd, Shanghai 200001, China

## Abstract

The gut microbiota is commonly referred to as a hidden organ due to its pivotal effects on host physiology, metabolism, nutrition and immunity. The gut microbes may be shaped by environmental and host genetic factors, and previous studies have focused on the roles of protein-coding genes. Here we show a link between long non-coding RNA (lncRNA) expression and gut microbes. By repurposing exon microarrays and comparing the lncRNA expression profiles between germ-free, conventional and different gnotobiotic mice, we revealed subgroups of lncRNAs that were specifically enriched in each condition. A nearest shrunken centroid methodology was applied to obtain lncRNA-based signatures to identify mice in different conditions. The lncRNA-based prediction model successfully identified different gnotobiotic mice from conventional and germ-free mice, and also discriminated mice harboring transplanted microbes from fecal samples of mice or zebra fishes. To achieve optimal prediction accuracy, fewer lncRNAs were required in the prediction model than protein-coding genes. Taken together, our study demonstrated the effecacy of lncRNA expression profiles in discriminating the types of microbes in the gut. These results also provide a resource of gut microbe-associated lncRNAs for the development of lncRNA biomarkers and the identification of functional lncRNAs in host-microbes interactions.

The intestinal tract harbors trillions of commensal bacteria representing over a thousand species and encoding over one hundred and fifty fold more genes than the human genome. The human intestinal microbiota has been shown to participate in epithelium maturation and proliferation, host nutrition and metabolism, as well as immune responses and protection against pathogens^1^,^2^. It is increasingly likely that specific compositional patterns of gut microbiota may associate with different human diseases, such as colorectal cancer^3^,^4^,^5^,^6^ and inflammatory bowel disease (IBD)^7^,^8^,^9^. The gut microbiota composition is shaped by multiple factors such as food intake, colonization history, and host genetic factors^10^,^11^. Our current knowledge about the relationship between host genetic background and microbiota composition is still limited, and most previous studies have focused on the potential roles of protein-coding genes^12^,^13^,^14^.

Recent genomic studies have revealed tens of thousands of long non-coding RNAs (lncRNAs) in the mammalian genomes^15^. LncRNAs may participate in many essential biological processes, such as genomic imprinting, maintenance of pluripotency, immune response and development. Moreover, lncRNAs have also been linked to different human diseases, such as neurodegenerative disorders, cardiovascular diseases and cancers^16^,^17^,^18^. While the roles of protein-coding genes in host-microbiota interactions have been subjected to intensive investigation, it is largely unclear if lncRNAs may participate in the responses of intestinal epithelial cells to gut flora.

Recent studies have suggested the involvement of lncRNAs in inflammatory signaling. As an example, lncRNA-Cox2 has been reported as a downstream target of TLR signaling that serves as a transcriptional cofactor through interactions with various regulatory complexes^19^. In addition, the lncRNA THRIL was found to regulate TNFα by binding to hnRNPL during innate activation of macrophages^20^. However, it is unknown whether and to what extent lncRNAs may be regulated by gut microbiota. It is also unclear if lncRNA expression profiles may reflect certain features of microbes in the gut.

In this study, we characterize lncRNAs that are regulated by gut microbiota (in conventional or gnotoboitic mice), which may be useful for further functional investigations. We also present a proof-of-concept study for the effecacy of lncRNA-based signatures in discriminating conventional and gnotobiotic mice.

## Results

### Identification of commensal microbiota-regulated lncRNAs

**Figure 1:**
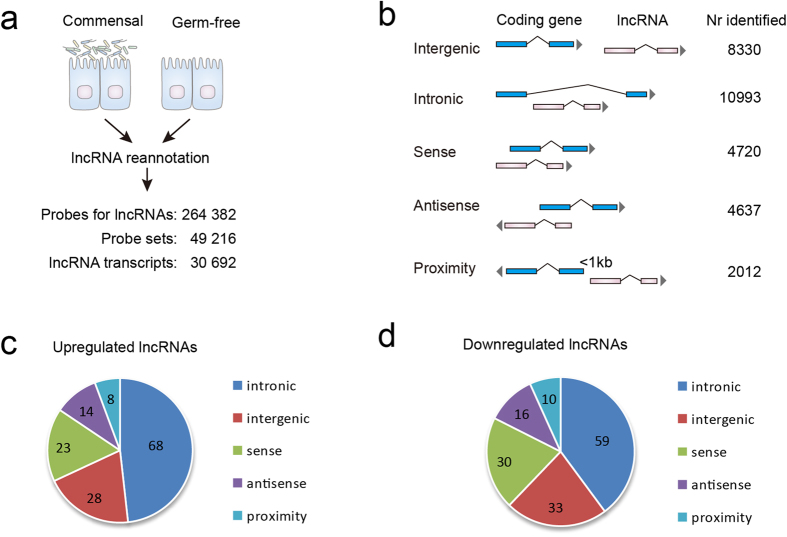
Identification of lncRNAs regulated by commensal microbiota in mice. (**A**) Schematic representation of the lncRNA reannotation process. The mouse exon array contained 264 382 probes mapping to 30692 lncRNA transcripts. Probes that overlap with coding genes have been removed. The dataset was based on intestinal epithelium cells of conventionalized mice as compared to germ-free mice. (**B**) The classification of lncRNAs into five categories (intergenic, intronic, sense, antisense and proximity) according to their relationships with protein-coding genes. The numbers of identified lncRNAs in different categories are shown on the right. (**C,D**) Pie charts showing the number of microbiota-regulated lncRNAs in each category. When a criteria of fold change>2 and P < 0.05 was accepted, 141 and 148 lncRNAs were respectively up- and down-regulated.

To systematically identify microbiota-regulated lncRNAs, we focused on published microbiota re-colonization studies that utilized the Affymetrix mouse exon microarray platform, which has many more probes mapping to lncRNA genes^15^,^21^. We were specifically interested in the lncRNA expression profiles in gut epithelial tissues that interact with gut microbes, thus the datasets concerning other tissues or cell types (e.g., liver, macrophages, etc) were excluded from further investigation. Since the gut microbiota of laboratory mice is variable due to both genetic and environmental factors^22^,^23^, we focused on data from one laboratory to avoid potential inconsistency caused by microbial variation. These criteria have selected the GSE46952 dataset^24^, which included conventional, germ-free and gnotobiotic mice (re-colonized with either E.coli or E.coli expressing bile salt hydrolase) with at least 4 biological replicates for each condition. A comprehensive computational pipeline^15^ was used to re-annotate the probes that uniquely map lncRNA transcripts (overall design shown in Fig. 1a). The reliability of this method has been supported by RT-PCR validation and the high consistency with RNA-seq data^15^. As indicated in Fig. 1b, five categories of lncRNAs have been identified according to their relationships with protein-coding genes, including intergenic, intronic, sense, antisense and proximity (a full list of lncRNAs is provided in Supplementary Table 1).

According to the findings of the MicroArray Quality Control (MAQC) project, fold-change based selection criteria can significantly improve the agreement of the biological interpretation of the data^25^. In contrast, when a t-statistic (P-value) ranking is used as the primary criterion the reproducibility would be substantially lower^26^. Therefore, we determined differentially expressed lncRNAs based on fold-change (>2 or <0.5) plus a nonstringent P-value cutoff (0.05). This criteria has been also been accepted in previous studies^27^,^28^. While intronic lncRNAs represented the largest group (35.8%) in all identified lncRNAs, we found even higher rates of intronic lncRNAs in both upregulated (48.2%, P < 0.001, chi-test) and downregulated lncRNAs (39.9%, P < 0.001, chi-test) caused by re-colonization of commensal microbiota in germ-free mice (Fig. 1c,d, altered lncRNAs listed in Supplementary Table 2).

### Limited overlap between lncRNAs associated with distinct gut microbes

**Figure 2:**
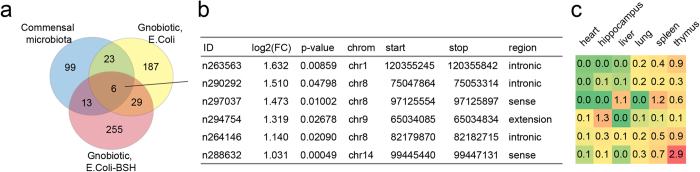
Venn’s diagram showing the overlapping lncRNAs that are induced by microbiota, E.coli or E.coli-BSH. The six commonly upregulated lncRNAs are described in the table, and their expression levels in different tissues (heart, hippocampus, liver, lung, spleen and thymus) are indicated in the heat map on the right. It can be seen that most lncRNAs are highly expressed in immune organs (spleen and thymus).

Since it has been suggested that gnotobiotic mice may have specific expression patterns of protein-coding genes^29^, we tested whether lncRNAs are also differentially expressed in mice that were re-colonized by different types of microorganisms. To this end, we analyzed the expression of lncRNAs in germ-free (GF) mice in comparison to mice that were re-colonized with either mouse microbiota (mouse), E.coli (EC) strain, or E.coli expressing bile salt hydrolase (EC-BSH). Interestingly, only low level of overlap was found between these conditions, with most altered lncRNAs being type-specific (Fig. 2a, listed in Supplementary Table 3). In the six commonly upregulated lncRNAs (Fig. 2b), most were also highly expressed in immune organs such as spleen and thymus (Fig. 2c), suggesting potential involvement of these lncRNAs in host immune responses.

**Figure 3:**
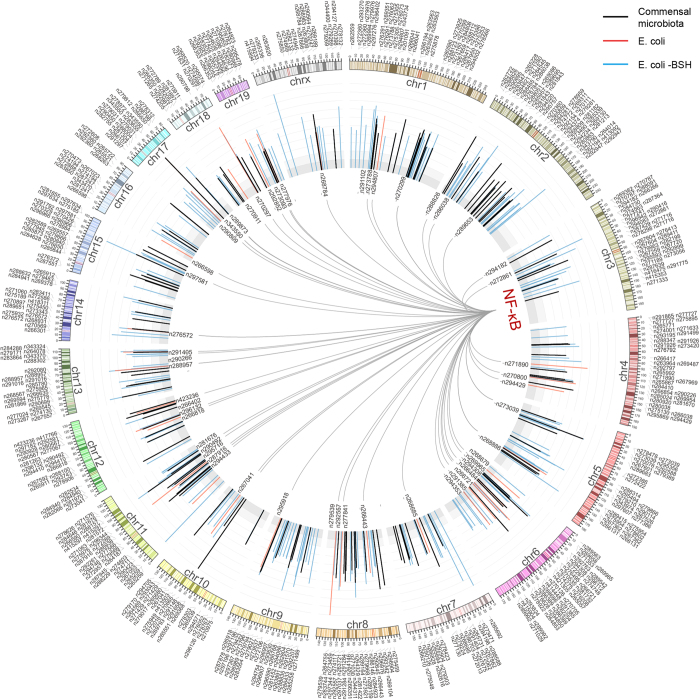
Circos plot showing the lncRNAs that are upregulated by commensal microbiota, Escherichia coli (E.coli), or Escherichia coli expressing bile salt hydrolase (E.coli-BSH). The histogram indicates expression levels of lncRNAs that are upregulated by microbiota (black), E.coli (blue) or E.coli-BSH (red). The links indicate potential binding of NF-κB to the promoters of these upregulated lncRNAs. The Genbank accession numbers of lncRNA transcripts are indicated in the plot.

Previous studies have demonstrated the crucial role of NF-κB in transactivating a large number of protein-coding genes in response to gut microbiota^1^,^30^,^31^, thus we explored the potential relationship between NF-κB and upregulated lncRNAs. The genomic binding sites of NF-κB was extracted from GSM611117 dataset, and were compared to the transcription starting sites (TSS) of upregulated lncRNAs. As a result, only 72 of 612 (11.7%) upregulated lncRNAs (in any condition) were found with NF-κB binding sites <10 kb upstream of their TSS (Fig. 3, listed in Supplementary Table 4), indicating that most upregulated lncRNAs may not be direct transcriptional targets of NF-κB.

### LncRNA-based signatures correctly identify gnotobiotic mice from conventional mice

**Figure 4:**
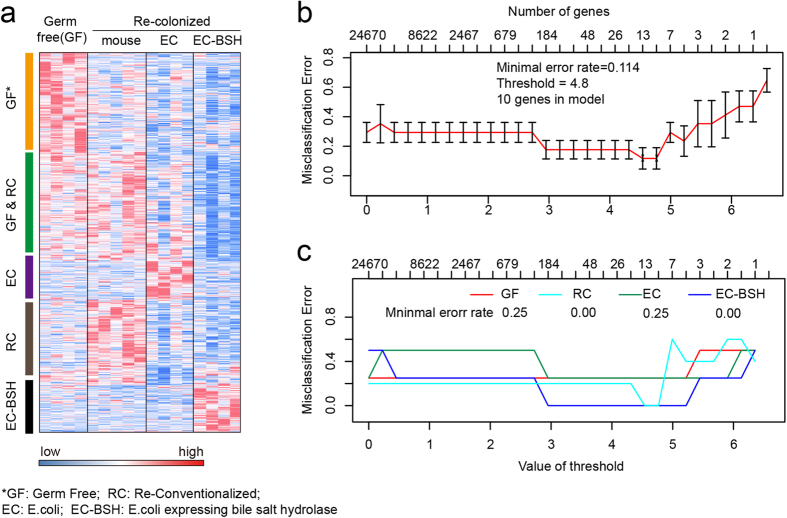
LncRNA expression profiles discriminate conventional and gnotobiotic mice. Four types of gut microorganism conditions in dataset GSE46952 (germ free, re-conventionalized with mouse microbiota, E.coli, or E.coli expressing bile salt hydrolase) were classified by PAM algorithm based on the expression profiles of lncRNAs. (**A**) The expression profiles of lncRNAs that have microbiota-specific expression in intestinal epithelium cells. The lncRNAs with greater centroids (>3) were selected by the PAM algorithm, which uses the nearest shrunken centroid methodology to identify sample types. These lncRNAs associate with specific microorganism status within the gut, such as germ free (GF), re-conventionalized mouse microbiota (RC), E.coli (EC), and E.coli expressing bile salt hydrolase (EC-BSH). (**B**) The misclassification error rates by PAM as a function of threshold for centroid shrinkage. The error rates firstly decreased at threshold of 3, and further reached minimal value (0.114) when threshold was set to 4.5. (**C**) Type-specific error rates for classification as a function of threshold for centroid shrinkage.

Although the exact mechanisms underlying microbiota-affected lncRNA expression are largely unknown, it is likely that lncRNA expression may partially result from host-microbe interactions and therefore constitute a signature that reflects the status in the gut. Based on this hyposis, we questioned if lncRNA expression profiles may provide sufficient information for discriminating gnotobiotic and conventional mice. Using an established method for feature extraction and sample classification named PAM algorithm^32^, we classified the germ-free (GF), re-conventionalized (RC), and gnotobiotic mice (EC or EC-BSH) based on lncRNA expression profiles. The PAM algorithm uses the “nearest shrunken centroids” model to identify gene signatures that best characterize each class, and its effectiveness on lncRNA-based sample classification has been demonstrated in our previous study^16^. As expected, the PAM algorithm identified lncRNAs with microbe-specific expression patterns (Fig. 4a), and the final classificiation model successfully discriminated these mice (GF, RC, EC and EC-BSH) with an overall error rate of 0.114 (Fig. 4b). Notably, the mice in EC and EC-BSH groups were classified without an error (Fig. 4c), suggesting that lncRNA expression profiles may discriminate gnotobiotic mice more efficiently from other types.

### LncRNA signatures discriminate mice with different transplanted microbiota

**Figure 5:**
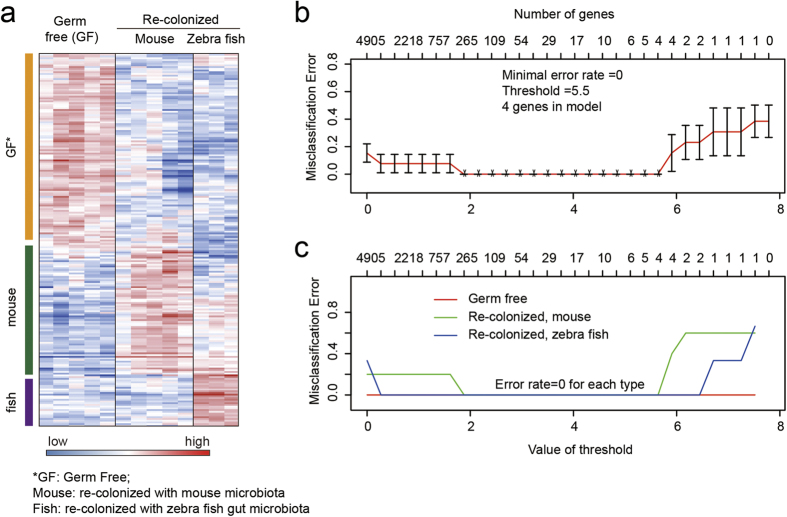
Discrimination of mice with gut microbiota transplanted from conventional mice or zebra fish. The lncRNA expression profiles were extracted from GSE5198 dataset, which included gene expression in the small intestine of mice that were either germ-free (GF), colonized with a conventional mouse cecal microbiota (Re-colonized, mouse) or colonized with a conventional zebrafish gut microbiota (re-colonized, zebrafish). (**A**) Heat map showing the sample-specific expression profiles of lncRNAs in intestinal epithelium cells. The genes were screened according to a centroid shrinkage threshold of 2, as determined by overall prediction error. (**B**) Overall prediction error rate as a function of centroid shrinkage threshold in the PAM classification model. An minimal error of 0.00 was reached when the threshold increased to 2. (**C**) Type-specific error rates for classification as a function of threshold for centroid shrinkage.

The higher accuracy for gnotobiotic mice identification was not surprising, since the colony of single bacterial strain may represent a status of extremely imbalanced microbiota. Therefore, it is meaningful to test if lncRNAs-based signatures could efficiently discriminate mice bearing composite microbes. The GSE5198 dataset included germ-free mice that were re-colonized with fecal-derived microbiota from mice or zebra fish. Interestingly, the PAM algorithm identified a considerable number of lncRNAs with type-specific expression (Fig. 5a), and perfectly discriminated all mouse groups (Fig. 5b,c). Therefore, it seems that lncRNA expression profiles could identify not only gnotobiotic mice, but also mice with different composite microbes.

## Discussions

Previous investigations have mainly focused on the potential roles of protein-coding genes in host-microbe interactions, but our results suggest a link between lncRNA expression and gut microbes. To probe the expression of lncRNAs in re-conventionalized and gnotobiotic mice, we used a comprehensive bioinformatics pipeline to reannotate probes that uniquely map to lncRNAs from public expression microarray datasets.

The comparisons between re-conventionalized (RC) mice and gnotobiotic mice (EC and EC-BSH) suggested considerable type-specific expression patterns of lncRNAs. Only six lncRNAs were commonly upregulated in RC, EC and EC-BSH mice, although 613 lncRNAs were found upregulated in at least one condition. These 6 lncRNAs were also highly expressed in immune organs such as spleen and thymus, suggesting their potential involvement in host immune responses. Since immune cells may be recruited and activated upon re-colonization of microbes in the gut, it still requires clarification whether these commonly upregulated lncRNAs may accurately reflect the change in epithelial cells alone.

Our classificaiton models based on lncRNA expression profiles have sucessfully discriminated mice that were re-colonized with different E. coli strains or fecal-derived microbiota. These findings further support a more generalized hypothesis that lncRNAs may be as important as protein-coding genes for the purposes of indicating biological status. As we have discussed previously, the expression level of a non-coding gene may better represent its activity than a protein-coding gene (PCG), because the function of a PCG may be affected by more factors such as translation, posttranslational modification, conformational regulation, and proteasomal degradation^17^,^18^.

To avoid potential inconsistency caused by microbial variation, our study was based on microarray data from the same laboratory. Future studies should test the cross-platform (e.g. RNA-seq vs microarray), cross-strain (BALB/c vs C57BL/6) and cross-laboratory overlapping of differentially expressed lncRNAs. It would also be worthwhile to further clarify the exact roles of lncRNAs in host-microbe interactions. Another important direction would be discovering disease-associated lncRNA signatures, which may be useful for developing novel biomarkers and therapeutic targets.

## Methods

### Re-annotation of microarray probes

The raw microarray data of mouse intestinal tissues were downloaded from Gene Expression Omnibus (GEO). The dataset GSE46952 included conventional mice (n = 5), germ-free (GF, n = 4), and gnotobiotic mice that were colonized with E.coli (EC, n = 4) or E.coli expressing bacterial bile salt hydrolase (EC-BSH, n = 5). The pipeline for annotating probes that uniquely map to lncRNAs has been described previously^15^. Briefly, the 4.7 million probes in the Affymetrix GeneChip Exon 1.0 ST arrays were filtered to discard those mapping to none or multiple locations, and probes overlapping with protein-coding genes were also excluded for further processing. The remaining probes were aligned with lncRNA genes (>200 bp) that were included in the NONECODE3 database^33^. After ambiguous hits were removed, the probes mapped to 30692 lncRNAs in the mouse genome.

### Identification of differentially expressed lncRNAs

The expression levels of lncRNAs were compared between different conditions using Linear Models for Microarray Data (LIMMA)^34^. A widely accepted criteria of fold change>2 and P < 0.05 was used to identify differentially expressed genes. According to the MicroArray Quality Control (MAQC) project^25^,^26^, gene lists generated by fold-change ranking plus a nonstringent P-value cutoff were more reproducible than those obtained by significance analysis. The genomic locations and expression levels of altered lncRNAs were visualized using the circos program^35^.

### Mapping NF-κB binding sites on lncRNA promoters

The NF-κB ChIP-seq data (on p65 subunit) were obtained from Expression Omnibus (GEO) with the accession number GSM611117. The distances between these peaks and the transcription starting sites (TSSs) of lncRNAs were calculated with the ChIPpeakAnno package of the Bioconductor program^36^. When the binding site was located within 10kb upstream the TSS of lncRNA, a putative binding was considered. This criteria has been adopted by many previous studies^37^,^38^,^39^.

### Sample classification based on gene expression profiles

We used lncRNA expression profiles to predict the types of mice, based on the PAM algorithm that shrinks the prototypes and hence obtains a classifier^32^. PAM applies the “nearest shrunken centroids” method to identify subsets of genes that best characterize each class. The shrinkage consists of moving the centroid towards zero by a threshold, which is determined according to the prediction error of the model. As the threshold increases, the number of genes left in the model decreases. In the present study, the prediction model was based on minimal sets of genes at a shrinkage threshold immediately before the error rates escalate. The prediction based on protein-coding genes used the same method as lncRNAs, and the threshold for centroid shrinkage was determined independently. Moreover, genes left in the model displayed strong type-specific expression feature when the increase of shrinkage caused an initial decrease in the misclassification error (note the threshold level was lower than the final value). In this context, genes left in the model displayed significant type-specific distribution patterns. The heat maps were generated using the TM4 software package^40^.

## Additional Information

**How to cite this article**: Liang, L. *et al.* Long noncoding RNA expression profiles in gut tissues constitute molecular signatures that reflect the types of microbes. *Sci. Rep.* **5**, 11763; doi: 10.1038/srep11763 (2015).

## Supplementary Material

Supplementary Information

Supplemental Table 1

Supplemental Table 2

Supplemental Table 3

Supplemental Table 4
