# Supplementary material for: Long noncoding RNA expression profiles in gut tissues constitute molecular signatures that reflect the types of microbes
Source: Sci Rep. 2015 Jun 30;5:11763. doi: 10.1038/srep11763 (PMC4485256; doi:10.1038/srep11763)
Supplement: Supplementary Information [file srep11763-s1.doc]

**Supplemental information**

**Title:**

Long noncoding RNA expression profiles in gut tissues constitute molecular signatures that reflect the types of microbes

**Author list:**

Lunxi Liang, Luoyan Ai, Jin Qian, Jing-Yuan Fang, and Jie Xu

**Supplemental Figure 1.** The expression profiles of protein-coding genes were used for prediction of mice that were either germ-free (GF), or re-colonized with conventional microbiota (RC), E.coli (EC) or E.coli expressing BSH (EC-BSH). The upper panel indicates the overall misclassification rate, and the lower panel shows the error rate for each sample type.


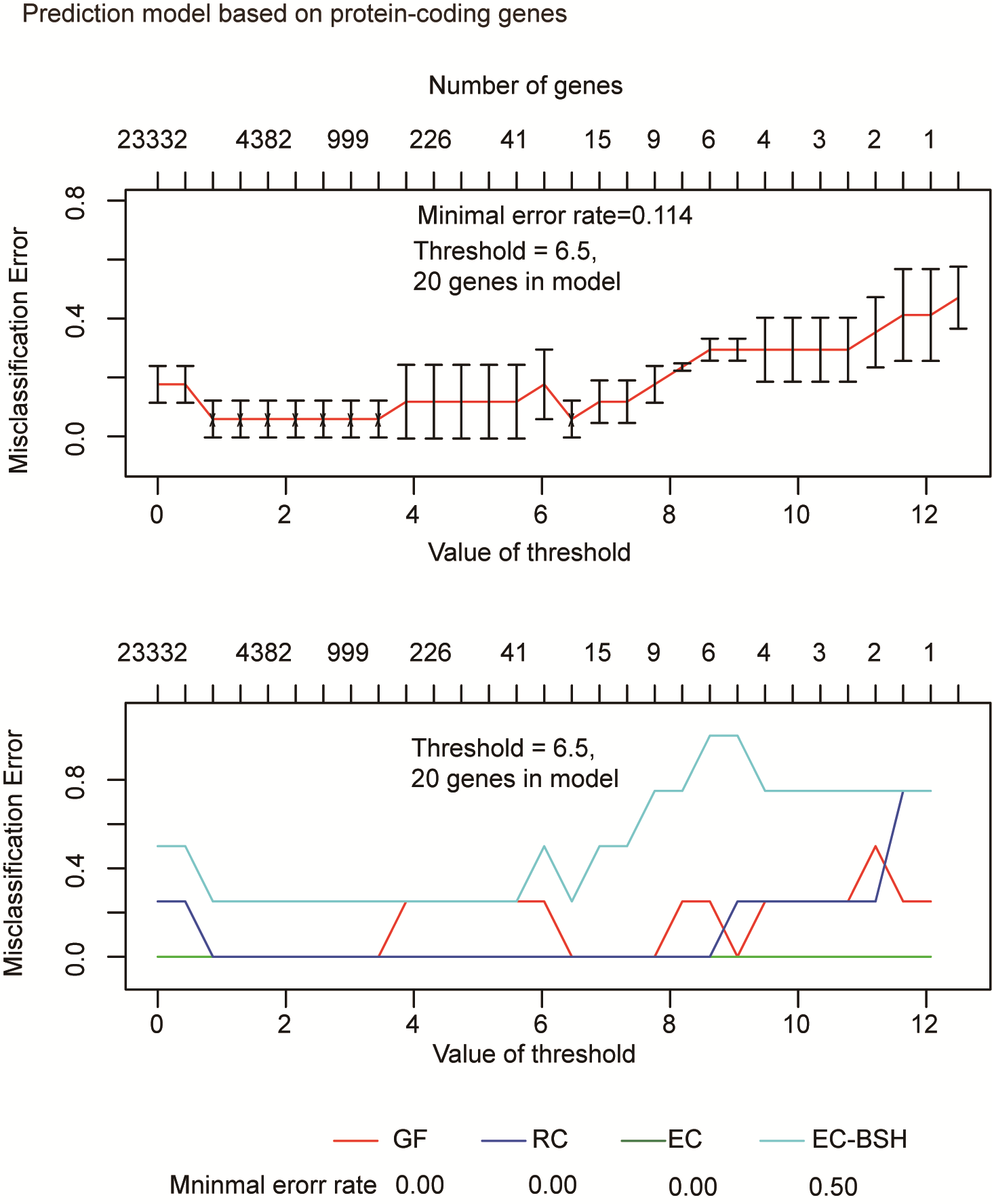


**Supplemental Figure 2**. The expression profiles of protein-coding genes were used for prediction of mice that were either germ-free, or re-colonized with the microbiota derived from fecal samples of mouse or zebra fish. The upper panel indicates the overall misclassification rate, and the lower panel shows the error rate for each sample type.


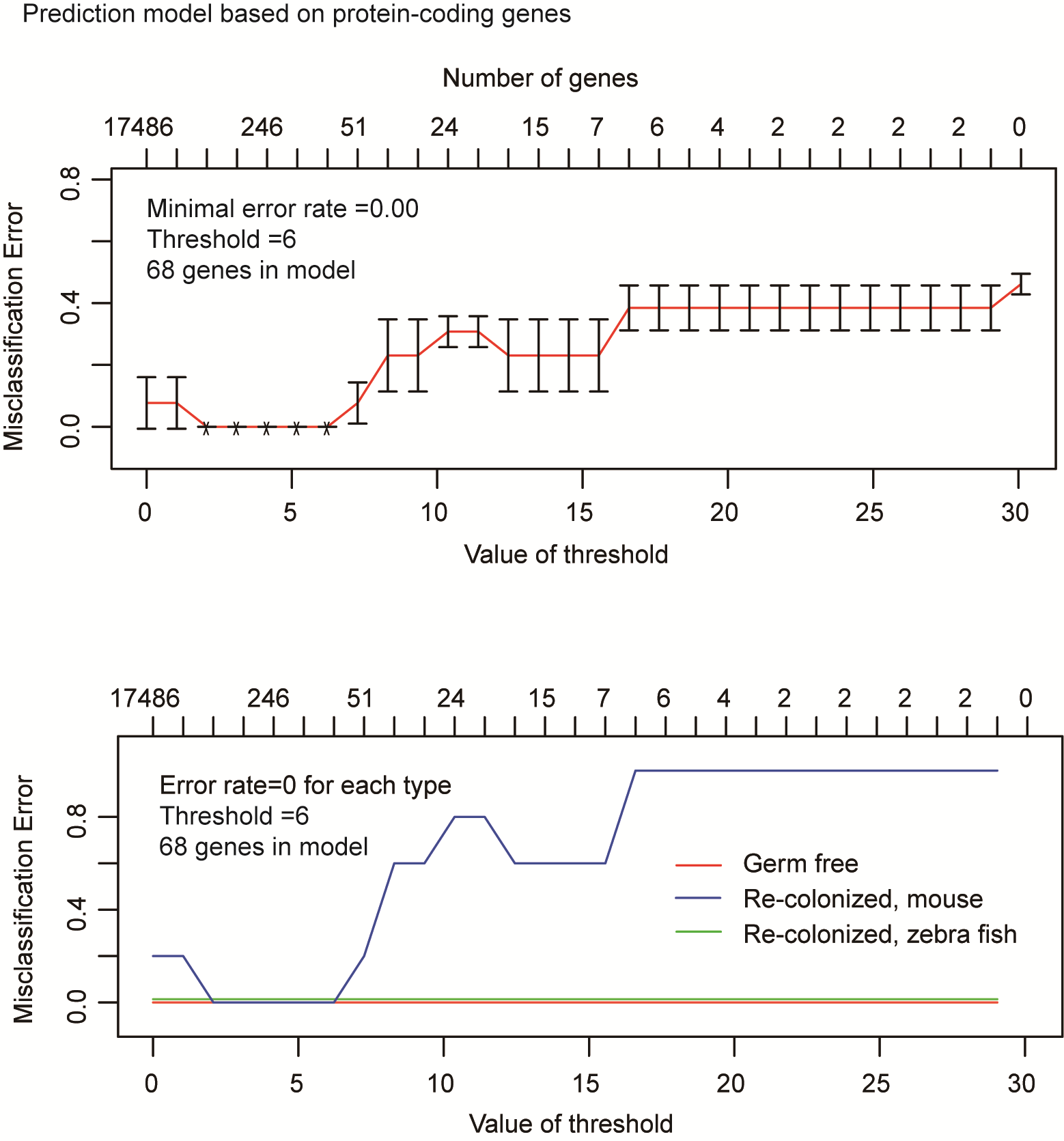


**Supplemental Table 1**. LncRNA gene table with fold changes and p-values. The fold changes (log2 transformed) of lncRNAs in re-conventionalized mice as compared to germ-free mice are shown in the second column.

**Supplemental Table 2**. Significantly altered lncRNAs in re-conventionalized mice as compared to germ-free mice. The lncRNAs are ranked in descent by their fold changes (log2 transformed).

**Supplemental Table 3**. Lists of upregulated lncRNAs by re-cloned microbiota, E.coli (EC) and E.coli expressing bile salt hydrolase (EC-BSH) are respectively shown in different tables.

**Supplemental Table 4**. Binding sites of NF-κB to the promoters of lncRNAs that were upregulated by either microbiota, EC or EC-BSH. The columns on the left describe the positions of lncRNAs in the genome, and the columns on the right indicate the features of ChIP-seq peaks by NF-κB (p65) pull down.
